# A mixed methods evaluation of a facilitated research career pathway for nurses, midwives, allied health professionals and healthcare scientists working in the NHS

**DOI:** 10.1186/s12909-025-07982-2

**Published:** 2025-10-17

**Authors:** Eleanor Lutman-White, Nicolas Aldridge, Ceri Jones, Agnieszka Lewko, Jane Coad

**Affiliations:** 1 Centre for Healthcare and Communities, Coventry University, Coventry, UK; 2 Research and Development, University Hospitals Coventry and Warwickshire NHS Trust, Coventry, UK; 3 School of Health Sciences, Faculty of Medicine and Health, University of Nottingham, Nottingham, UK

**Keywords:** Research culture, Research capacity, Clinical academic careers, Nurses, Midwives, Allied health professionals

## Abstract

**Background:**

Opportunities for nurses, midwives, allied health professionals and healthcare scientists (NMAHP+s) to engage in research are increasing but still lag behind opportunities for medical staff.Iinitiatives to identify, support and develop NMAHP+s considering and pursuing clinical academic roles are key to growing an NMAHP+ clinical academic workforce. We describe and evaluate the impact of the Interdisciplinary Clinical Academic health Research Excellence facilitated research career pathway (iCAhRE^™^ programme) on NMAHP+ participants in one National Health Service Trust in England.

**Methods:**

The study used a mixed methods approach informed by the Kirkpatrick Model of Training/Evaluation. Quantitative data related to uptake, progression through programme stages and key demographics of participants in the programme. Twenty-nine participants participated in a survey which assessed experiences of the programme, learning and impact from the programme, career trajectories, and barriers and enablers to pursuing a clinical academic career. Experiences of the programme were captured in greater depth in 12 interviews.

**Results:**

Since 2016, participants were able to engage with the programme appropriate to their needs and research careers. Responses to the programme were highly positive and participants had increased their research knowledge and skills, initiated new research-related activities, improved their practice and increased their confidence. A high number had progressed to a role with a higher banding compared to when they started the programme (37.9%) and had research as part of their current role (55.2%). However, significant challenges to pursuing a clinical academic career for NMAHP+s included: unsupportive managers, lack of dedicated time for research engagement in a context of clinical pressures, a lack of clinical academic posts and limited progression opportunities.

**Conclusions:**

This NHS Trust-supported high-quality facilitated research career pathway has lasting effects, increasing research knowledge and skills, informing and changing participants’ clinical practice, building confidence, increasing research-related activities undertaken and career progression. The programme’s range of opportunities across the whole pathway, the strong collaborative partnership between the NHS Trust and partner Higher Education Institute (HEI) and visible support for research are important contributors to the programme’s impact. However, challenges to pursuing a clinical academic career for NMAHP+s persist and require further attention.

**Supplementary Information:**

The online version contains supplementary material available at 10.1186/s12909-025-07982-2.

## Background

In healthcare, research engagement by clinicians and organisations is central to ensuring that patients receive high quality care and better outcomes. There is evidence that research active healthcare organisations have lower risk-adjusted mortality rates, improved processes and outcomes of care, and higher levels of patient experience [1–3]. The importance of research and innovation for improving the health and care of the population is identified in the NHS Long Term Plan [4] and its Constitution [5].

Developing a sustainable and supported research workforce, including development of clinical academics (healthcare professionals who work within and across both clinical and academic environments [6]) is recognised as fundamental to achieving this impact [7, 8]. There have been fewer opportunities to engage in research for nurses, midwives, allied health professionals and healthcare scientists (NMAHP+s) compared to medical staff as clinical academic career pathways in medicine are well-established, resourced and have a standardised UK trajectory [9]. NMAHP+s form the vast majority of the clinical workforce which provides care to patients [6], yet inequalities in access to clinical academic career pathways means that they are not at the same stage of research capacity development as medicine. Numerous workplace barriers also exist, such lack of time [10–12], lack of resources [11], lack of research knowledge, experience and expertise [11] and lack of organisational and management support [13–15], preventing NMAHP+s realising ambitions to engage in research activity.

Within the UK, research capacity and capability building in NMAHP+ groups is a clear ambition within policy frameworks from the Chief Nursing Officer in England [16], The Royal College of Midwives [17] and Health Education England [18] for Allied Health Professionals. Opportunities for NMAHP+s increased over the past 15 years since the establishment of the Health Education England (HEE)/National Institute for Health and Care Research (NIHR) Integrated Clinical Academic and Practitioner Programme in 2008. However, these schemes are not available across the UK and in England are highly competitive. Therefore overall, this is insufficient to grow a significant body of NMAHP+ clinical academics. There is a need for local initiatives to identify, support and further develop NMAHP+s considering and pursuing clinical academic roles outside of these schemes. Local initiatives can be specific to a career stage, a type of research training or an element of career development or they can span the whole pathway [14]. There is increasing evidence around the implementation and evaluation of locally and regionally based programmes developing clinical academic career pathways [19–24], but the longer-term outcomes and impacts are frequently absent from evaluations [25] and evaluations of whole pathway approaches are lacking.

Responding to this gap, this paper aims to describe and evaluate the interdisciplinary Clinical Academic health Research Excellence (iCAhRE) facilitated research career pathway offered to NMAHP+s in one NHS Trust (Hospital organisation – acute and community) in England since 2016, focusing on outcomes related to programme participants’ personal and professional development.

### The iCAhRE programme

The iCAhRE programme is an ambitious research capacity and capability building programme developed by University Hospitals Coventry & Warwickshire NHS Trust (UHCW) to support the trajectory of staff careers in order to improve outcomes and benefit to patients and their families. The initial vision and prototype programme (Interdisciplinary Non-medical Clinical Academic or INCA) was developed in 2016 and was re-developed and re-launched as iCAhRE in 2019. It actively supports staff to pursue clinical academic research careers and those who want to gain a greater understanding of clinical research and the place it has in a modern health service. The programme is delivered at no cost to the staff who attend. It specifically focuses on providing opportunities for NMAHP+s, recognising that whilst integrated clinical and research career pathways are well established for doctors, comparable opportunities are less available for other professional groups.

The initial programme was jointly developed by two organisations and continues to be refined in partnership with Coventry University, arising out of the long-term collaborative relationship between the NHS Trust and the Higher Education Institution (HEI). Key members of the programme’s leadership team, a clinically focused university professor and the NHS Trust Head of Research and Development (R&D), have remained consistent since the inception of the programme. The programme’s approach has been aligned from the outset with the NHS Trust’s Organisational Strategy which puts patients first and aims to be a national and international leader in healthcare with involvement in cutting-edge research and innovation being central to this. There has also consistently been strong senior leadership support for the programme at the NHS Trust.

#### Programme aims

The overall strategic aims of the iCAhRE programme are:

1. To develop capacity and capability^1^ through developing and embedding clinical academic research careers and roles across interdisciplinary groups in the NHS Trust
2. To develop opportunities for research training and education for clinical academic researchers.
3. To develop a supportive and empowering infrastructure alongside embedding an innovative interdisciplinary clinical academic research culture across the NHS Trust.

#### Programme structure

**Fig. 1:**
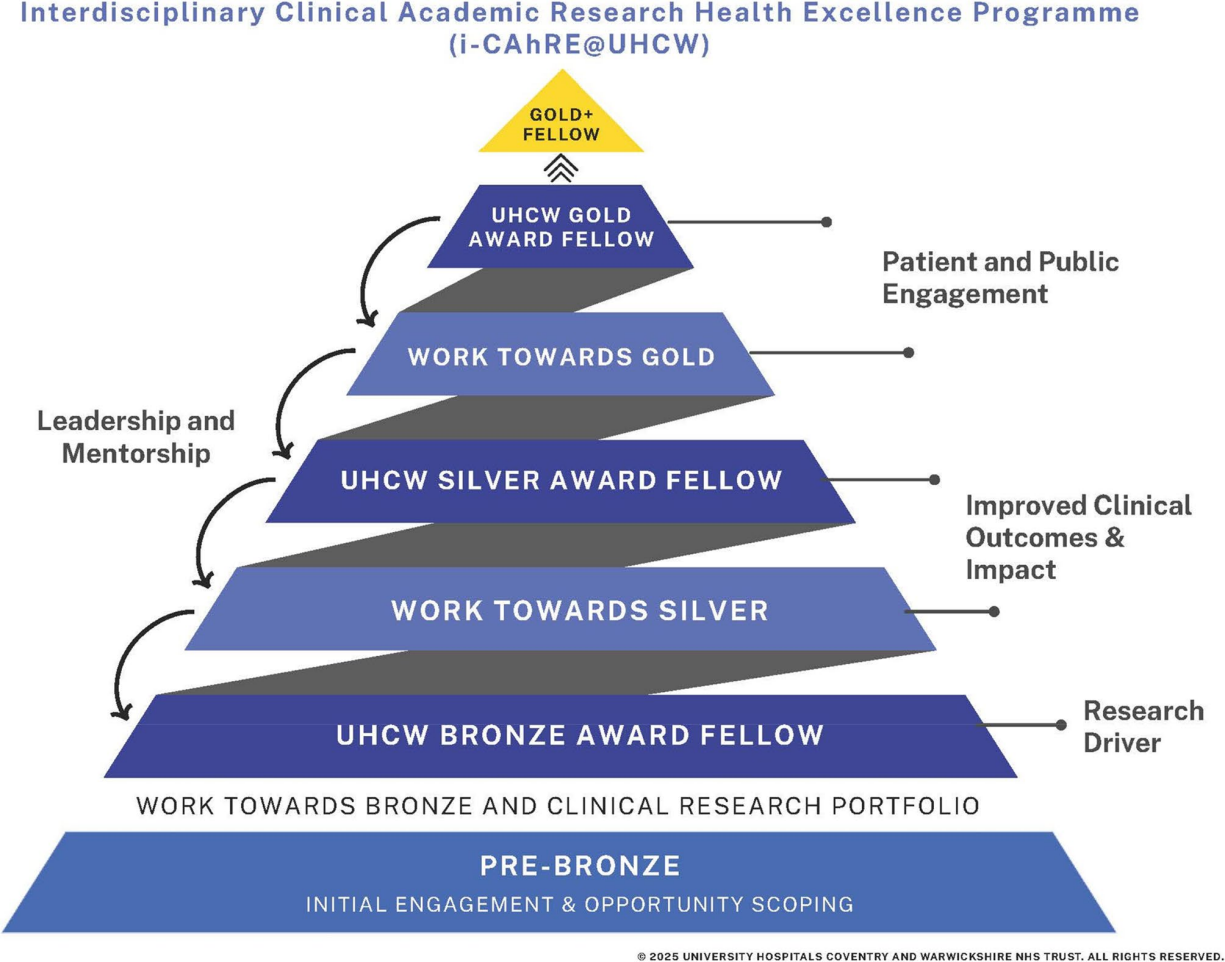
Levels of the iCAhRE programme

In line with national programmes for clinical academic researchers (e.g. NIHR Integrated Clinical Academic and Practitioner (ICA) Programme established in 2008), iCAhRE encompasses different levels that build research capacity and capability (see Fig. 1). It takes staff from graduation at degree level (known as Pre-Bronze) through to post-Doctorate level (known as Gold Plus), supporting access to academic and research training awards (e.g. HEE/NHIR ICA) whilst developing research skills and capabilities that enhance clinical practitioner competencies. At every stage mentoring and leadership is built in. In addition, support is offered in between levels. The programme is delivered and supported by staff ranging from administrators to senior academic professors from the NHS Trust and the local partner HEI, Coventry University.

iCAhRE Pre-Bronze** –** This includes identification of talented staff and supportive career discussions. The focus is on staff who have a degree related to healthcare and who demonstrate an interest in and understanding of research. Staff are supported in attending relevant opportunities within the NHS Trust and nationally and are added to R&D newsletters and informed of any upcoming relevant opportunities.

iCAhRE Bronze** –** This includes theoretical understanding for clinical research at an ‘introductory’ level to support and prepare clinical non-medical professionals to apply for a Master’s in Research or similar programme. It includes a stand-alone research-based university module and real life understanding of clinical research using research placements and clinical research supervision to support the development of research ideas. The module is delivered by Coventry University once or twice per year to a cohort of around 6–10 learners. The time commitment is 14 days over 3–4 months. This module gives staff an opportunity to find out more about research and whether a research career might be for them. There is no obligation to continue with any academic training beyond completion of the module. However, the aim of the Bronze programme is to encourage staff to look for opportunities and develop their clinical research career in the future.

iCAhRE Silver** –** This includes theoretical understanding for clinical research at a Master’s pre-doctoral level to support and prepare staff during and post-qualifying in a Master’s in Research or similar programme. Time commitment depends on whether the participant’s choice of Master’s is part or full time. The participant is allocated a supervisory team as part of their Master’s programme. In addition, within the NHS Trust iCAhRE Silver participants have access to range of experts within their clinical teams, one to one meetings with senior academics, drop-in clinics led by HEI associate professors/professors and access to a dedicated senior academic professor (Coad). The NHS Trust sends regular bulletins detailing funding and fellowship opportunities to iCAhRE Silver participants.

iCAhRE Gold** –**This level supports and prepares NMAHP+ professionals during a doctoral programme delivered by a HEI provider. Time commitment from participants is commensurate with whether the PhD programme is part or full-time. Within the PhD programme, participants have a supervisory team and meet three times a year with a dedicated senior academic professor (Coad) to discuss progress, career planning and facilitation of development opportunities. The NHS Trust sends regular bulletins detailing funding and fellowship opportunities to iCAhRE Gold participants.

iCAhRE Gold**+ –** if staff have attained PhDs, one to one mentoring is provided by a senior academic professor (Coad). This includes writing successful grants and project management, supervision of PhD students, supporting Gold+ Fellows to supervise Bronze and Silver Fellows and over-seeing of quality outputs such as presentations and articles.

The programme aims to provide an offering that is tailored to an individual’s needs. As well as participating in levels of the programme appropriate to each person’s planned career pathway, the programme also offers a wide range of other opportunities (see Table 1.). In addition, part of the agreement is the concept of ‘receiving and getting back’ as programme participants develop their own clinical academic research, in return they mentor others.

**Table 1.** Opportunities offered within the iCAhRE programme

**Opportunities offered within the iCAhRE programme are offered regularly (at least every two months) and include (but are not limited to):**
• Writing workshops
• Library support
• Journal clubs
• Support around governance
• Opportunities for Patient and Public Involvement and Engagement
• Support for applying for external funding opportunities – internal peer review
• Signposting to relevant opportunities
• Interview practice
• Shadowing opportunities
• Research placements
• Opportunities for networking and connecting with other staff

Some elements of the programme (e.g. the Bronze module) were paused for a period during the Covid-19 pandemic. More recently, the programme now forms part of R&D’s ‘Research for All’ strategy which aims to open up research opportunities to all staff within the NHS Trust. It is also now offered regionally to other NHS Trusts within the region.

### Aim and objectives of the study

This research aimed to evaluate the iCAhRE programme to assess reach, learner experiences, delivery outcomes, facilitators and barriers to engagement, and future opportunities, providing recommendations for improvements to the programme which could also inform future similar course development. The evaluation focused on the learning journey of the individual and the outcomes related to their personal and professional development; evaluation of economic benefit and return on investment was outside its scope.

## Methods

### Design

**Fig. 2:**
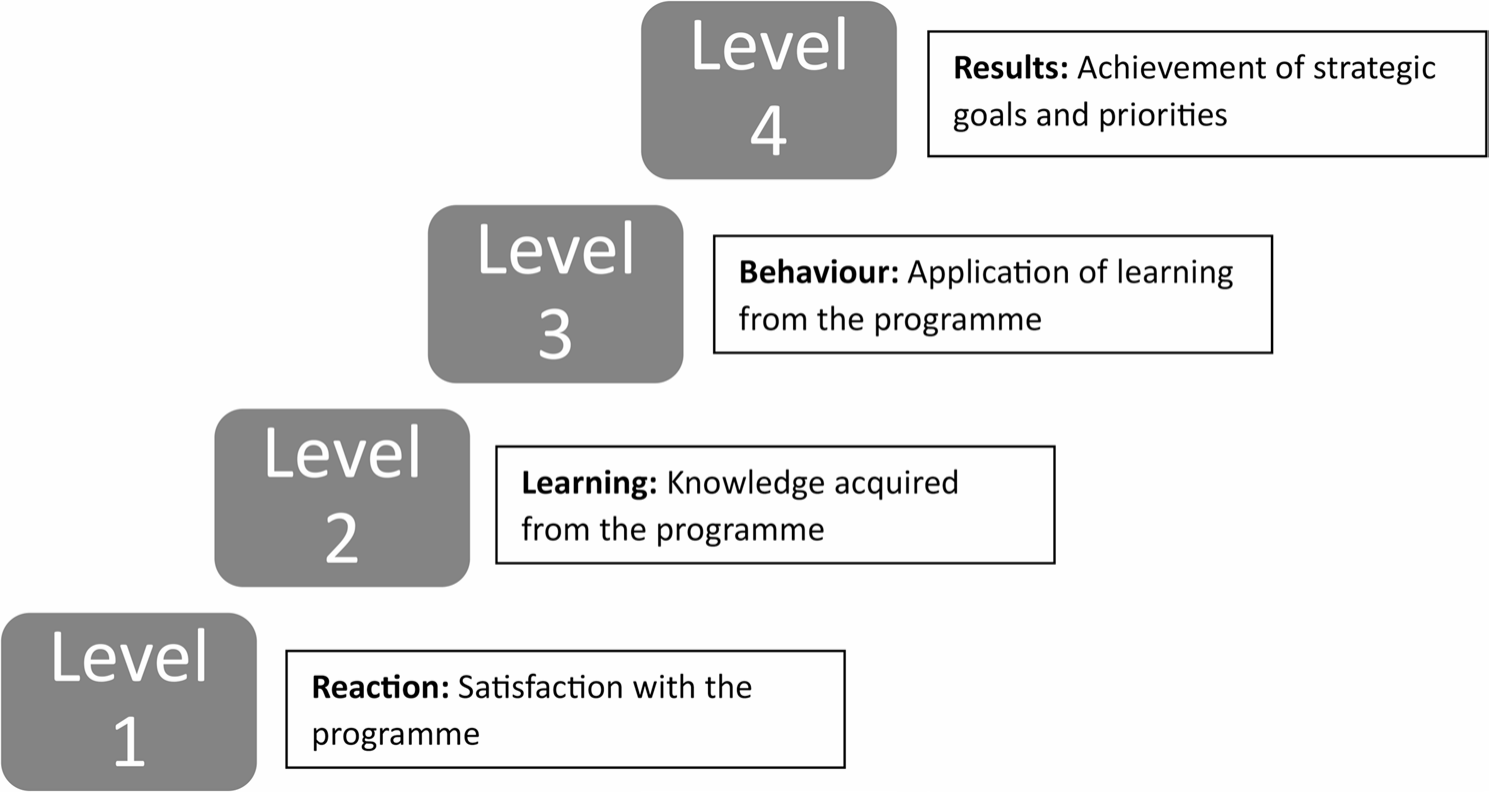
Kirkpatrick’s framework

The evaluation of iCAhRE employed a mixed methods design informed by Kirkpatrick’s four-level Evaluation Model [26, 27] to examine results and impact in relation to programme participants’ personal and professional development and organisational priorities (see Fig. 2). The model determines participants’ reactions in relation to their satisfaction with the learning event (level 1). Learning (level 2) relates to the extent to which participants perceived the intended knowledge, skills, attitude, confidence and commitment were acquired. Behaviour (level 3) is whether participants applied what they had learnt in the programme. Results (level 4) is the achievement of targeted outcomes following programme participation. An additional area of focus, the reach of the programme, was also included in this evaluation.

The study had three data gathering components enabling generation of key data against the four levels of Kirkpatrick’s framework. Routinely collected NHS Trust data on the reach of the programme and key programme outcomes comprised the first stage of the study. In the second stage a questionnaire enabled feedback about the programme, application of learning, impact of the programme and outcome data to be gathered, with opportunities for free text responses. The third stage of the study used qualitative methodology to enable deeper exploration of experiences from the perspective of programme participants.

### Quantitative data

Routinely collected NHS Trust anonymous data were analysed. This included data related to programme uptake since programme commencement in 2016, progression through programme stages, research activity (such as publications and research funding) between 2016 and 2024 and demographic information about programme participants.

### Survey

An anonymous online survey was developed for this study (see supplementary file 1) and administered via Qualtrics. Embedded within the questionnaire was full study information, a privacy notice and explicit informed consent. Consent was required before progressing to the main questionnaire. Participants were free to exit the questionnaire at any point by exiting and closing their browser. Survey responses were anonymous unless the participant provided their contact details for an interview. Participants who wished to submit their survey responses anonymously and take part in an interview were given the option to email the research team separately about an interview.

A purposive sample of current and former participants in the iCAhRE programme was identified by a Steering Group comprised of key members of the Research & Development (R&D) and iCAhRE teams at the NHS Trust and invited to participate in the online survey.

The design of the survey was based on the four levels of the Kirkpatrick Evaluation Model and comprised seven sections. This included questions about current role and profession; the degree to which participants found the programme favourable, engaging and relevant; their learning from the programme and the degree to which they had applied what they had learnt. Participants were also asked for demographics of age, gender identity, ethnicity, disability and caring responsibilities which were optional to report. Single and multiple choice questions, as well as the Likert scale to evaluate satisfaction, were used. Open-ended questions were also included so participants could provide further comments. The survey was piloted by research team members and amended before distribution to participants. The survey opened on 19th February 2024 and the final submission was on 16th April 2024.

### Interviews

All survey respondents were offered the opportunity to share their views in more depth in a semi-structured interview. An interview guide based on the four levels of the Kirkpatrick Evaluation Model was developed for this study (see supplementary file 2). Questions covered decision making around applying to commence the programme, experiences of the programme (including progression through the levels where appropriate), learning and impact from the programme, career trajectory, career aspirations and barriers and enablers to pursuing a clinical academic career. Participants were reminded that their responses would remain anonymous upon analysis and write-up. Consent to take part in interviews was provided online via Qualtrics. The online (via Teams) or in person interviews took place between March and May 2024 and were audio recorded. Interviews were conducted by an experienced, female qualitative researcher (ELW), who did not have experience of being a clinical academic but who has experience of developing a research career. Participants were reminded that the interviewer was not involved in the development or delivery of iCAhRE to reassure participants that honest disclosure of their opinions was encouraged.

### Data analysis

Quantitative data provided by the NHS Trust and from the survey were analysed using descriptive statistics in SPSS 28. Comparisons between organisation-level data and the programme participant data used two-samples tests of proportions in StataMP 15. Free text survey responses were analysed thematically either alone or alongside the interview transcripts where relevant. Together with qualitative data from the survey, interviews were anonymised, transcribed, and then coded for emerging themes using NVivo 14 by ELW. A second researcher (NA) also did initial coding of four interviews to check alignment of codes between researchers and ensure transparency of the analysis. The analytic process adopted a reflexive thematic analysis approach described by Clarke and Braun [28] using a hybrid of deductive and inductive coding. The data were coded deductively into the four levels of the Kirkpatrick Evaluation Model. Any utterances that were relevant to the research question but did not align with the Kirkpatrick Evaluation Model were coded inductively. Quotes from both the interviews and survey free text responses illustrate the themes.

Methodological triangulation combined survey and interview findings to provide an overall representation of participants’ responses in line with each level of the Kirkpatrick Evaluation Model plus an additional theme on barriers and enablers to progressing clinical academic careers. Qualitative and quantitative findings were largely complementary, with qualitative interview data found to agree with, and expand upon, survey responses.

## Results

### Reach of the programme

**Fig. 3:**
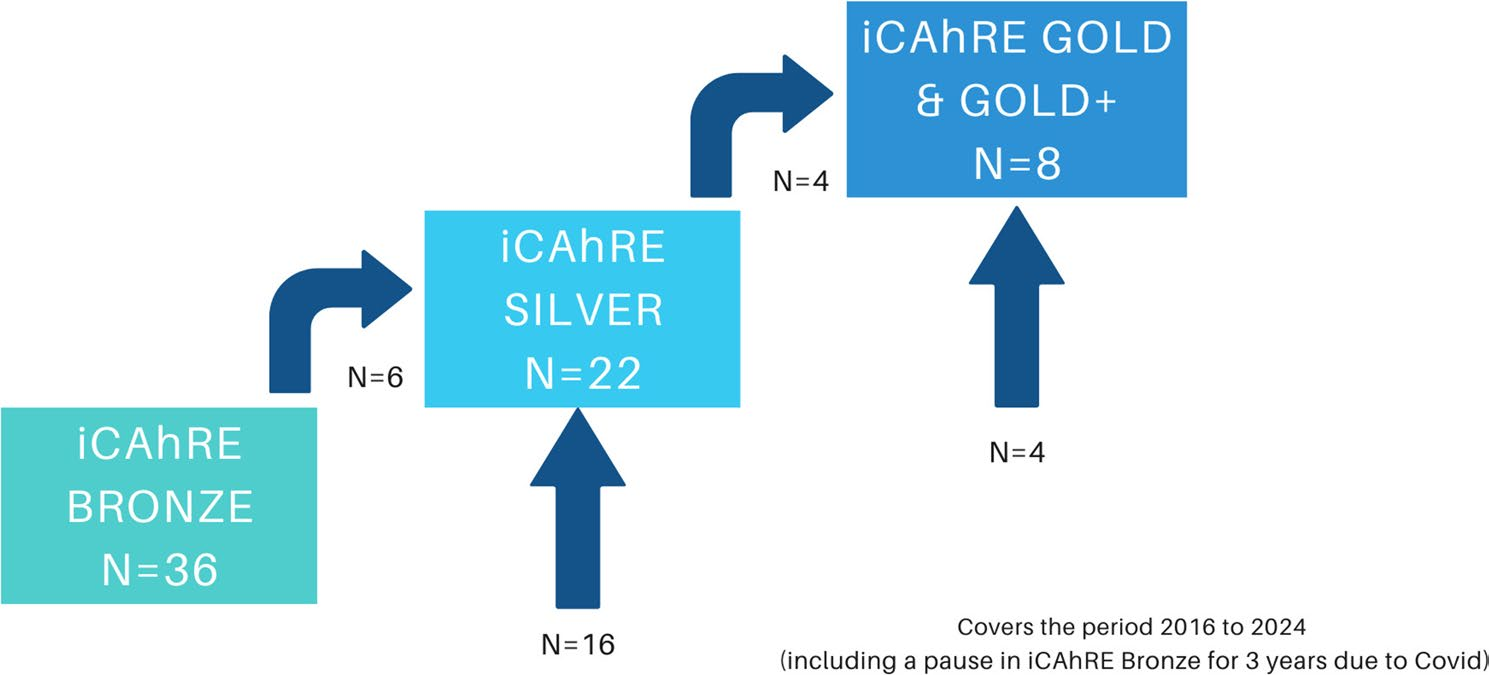
Flow of participants through the iCAhRE programme

Between 2016 and 2023, 36 staff members completed Bronze. There were 22 staff members who participated in Silver, six of whom had participated in Bronze. Eight staff members were involved in Gold and Gold+, four of whom completed Silver. Staff members who already had research Master’s degrees or PhDs entered the programme at Gold/Gold+ level. Two staff members had progressed through the whole pathway from Bronze to Gold/Gold+. The flow of participants through the pathway is shown in Fig. 3.

Nurses were the largest professional group to participate in Bronze and Sliver (see Table 2). When compared to the proportions among all NHS Trust NMAHP+s employees, nurses and midwives were underrepresented (z = 2.83, *p* = 0.005) and AHPs overrepresented (z=−3.271, *p* = 0.001) within Bronze, Silver and Gold/Gold+ participants (two-sample test of proportions). More females than males participated in all levels of the programme and these proportions were not significantly different from the proportions of male and female NMAHP+ employees in the NHS Trust.

**Table 2:** Demographic characteristics of Bronze, Silver and Gold/Gold+ participants

	Bronze (*n* = 36) *n* (%)	Silver (*n* = 22) *n* (%)	Gold/Gold+ (*n* = 8) *n* (%)
Professional group			
Nurse	12 (33.3)	7 (31.8)	1 (12.5)
Radiographer	7 (19.4)	1 (4.5)	-
Physiotherapist	3 (8.3)	3 (13.6)	1 (12.5)
Dietician	2 (5.6)	4 (18.2)	2 (25)
Healthcare scientist	2 (5.6)	1 (4.5)	2 (25)
Midwife	1 (2.8)	4 (18.2)	2 (25)
Psychologist	1 (2.8)	1 (4.5)	-
Not a registered healthcare professional	2 (5.6)	-	-
Missing data	6 (16.7)	1 (4.5)	-
**Gender**			
Female	24 (66.7)	16 (72.7)	6 (75)
Male	10 (27.8)	5 (22.7)	2 (25)
Missing data	2 (5.6)	2 (9.1)	-

Individuals may appear in this table more than once as some completed more than one level of the programme

### Evaluation participant characteristics

#### Survey participants

Twenty-nine programme participants completed the survey, a response rate of 38% (29/76 individuals who had taken part in Bronze/Silver/Gold/Gold + or had received support from the programme outside of these levels e.g. support for applying for funding opportunities). Survey respondents had participated in different aspects of the programme representing a range of experiences of different levels of the programme. Most respondents still worked at the NHS Trust (20 of 26; 76.9%). The largest groups of respondents were Allied Health Professionals (37.9%) and Nurses/Midwives (37.9%). Survey sample characteristics are presented in Table 3. 

**Table 3:** Survey participant characteristics

	Participants ( *n* = 29) *n* (%)
Professional group	
Allied health professionals	11 (37.9)
Nursing and midwifery	11 (37.9)
Healthcare scientists	2 (6.9)
Additional clinical services	1 (3.4)
Not reported	4 (3.4)
**Years of post qualifying/registration experience**	
0–5	2 (6.9)
6–10	2 (6.9)
11–15	4 (13.8)
16–20	6 (20.7)
21–25	7 (24.1)
30+	4 (13.8)
Not reported	4 (13.8)
**Internationally trained**	
Yes	9 (31)
No	14 (48.3)
Not reported	6 (20.7)
**Employment type**	
Part-time	4 (13.8)
Full-time	20 (69)
Not reported	5 (17.2)
**Age (years)**	
21–30	2 (6.9)
31–40	6 (20.7)
41–50	9 (31)
51+	6 (20.7)
Not reported	6 (20.7)
**Gender**	
Female	16 (55.2)
Male	7 (24.1)
Not reported	6 (20.7)
**Ethnicity** ^1^	
White English, Welsh, Scottish, Northern Irish or British	13 (44.8)
White Irish	1 (3.4)
Any other White background	2 (6.9)
Any other mixed or multiple ethnic background	1 (3.4)
Indian	2 (6.9)
Black African	2 (6.9)
Any other Black, African or Caribbean background	1 (3.3)
Not reported	7 (24.1)
**Disability**	
Yes	0
No	22 (75.9)
Not reported	7 (24.1)
**Parental/caring responsibilities**	
Yes	14 (48.3)
No	10 (34.5)
Not reported	5 (17.2)

^1^ Only ethnic groups selected by at least one respondent are reported here.

#### Interview participants

nts who had completed the survey and were representative of survey participants in terms of characteristics. The interviews lasted between 24 and 56 min (mean 41 min).

### Satisfaction with the iCAhRE programme (Kirkpatrick level 1)

**Fig. 4:**
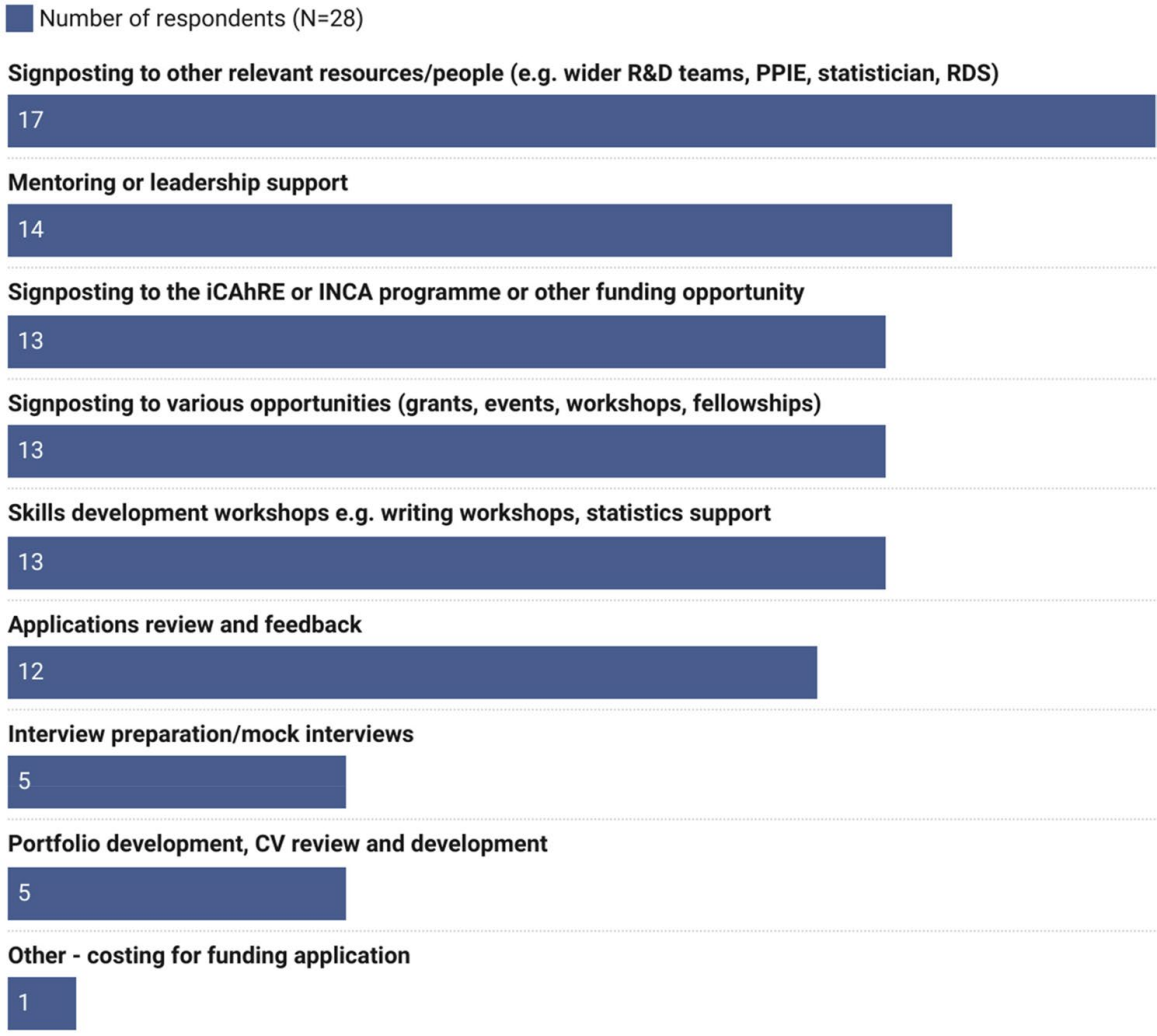
Support received from the iCAhRE programme

The programme offers a range of specific types of support for participants and survey respondents had accessed multiple types of support. Seventeen survey respondents reported that they had been signposted to relevant resources and people (e.g. wider R&D team, Patient and Public Involvement, statistician). Interview preparation/mock interviews and portfolio development/CV review were accessed less frequently. See Fig. 4.

On a scale of 1 to 10 (very dissatisfied to extremely satisfied) median (IQR) overall satisfaction with the opportunities offered through the programme was 8 (3). Eighteen survey respondents (62.1%) gave a rating of 8 or above. Eighteen survey respondents (62.1%) gave a rating of 8 or above.

Survey respondents reported on levels of satisfaction with specific aspects of the programme. Almost all participants (24 of 25, 96%) who responded to the question enjoyed the programme. 84% of survey respondents (21 of 25) agreed that the programme considered their individual journey. Almost 90% of participants (23 of 26, 88.4%) would encourage colleagues to seek support from the programme as a first option and the same proportion were clear about the iCAhRE programme’s purpose and strategy. 85% of survey respondents (22 of 26) agreed that their awareness of research-related opportunities such as grants, events and fellowships had increased due to involvement with the programme. However, there were aspects of the programme where there were lower levels of satisfaction. Less than half of respondents felt that the NHS Trust had enough research development opportunities for staff (12 of 26, 46.2%). 15% of survey respondents (4 of 26) felt that managers at the NHS Trust were not supportive of research and almost two-fifths (10 of 26, 38.4%) disagreed or were neutral about whether iCAhRE was essential for developing a research career.

Open-text responses from the survey and qualitative interview data provided additional detail on what participants liked about the programme. Generally, they liked that the Bronze module gave a broad overview of research methods and the research process which enabled participants to develop their interests further if they wanted to. Participants were particularly complimentary about the staff involved in organising and delivering the programme.

*The iCAhRE team were very approachable*,* supportive*,* and helpful. They were also very personable and friendly - this accessibility was very important so it didn’t seem so daunting approaching the R&D team as a (at the time) new internationally-educated nurse at UHCW. (Open text survey response)*

However, there were areas of the programme where participants suggested improvements. Some wanted more opportunities to learn and develop writing skills, more input in relation to applying for research funding and greater opportunities to connect with other iCAhRE participants.

On a scale of 1 (would not recommend) to 10 (would definitely recommend) indicating the extent to which survey respondents would recommend the programme to others, median (IQR) extent of recommendation was 9 (2). Twenty-two survey respondents (79.5%) gave a rating of 8 or above.

### Learning acquired from the iCAhRE programme (Kirkpatrick level 2)

The acquisition of specific research knowledge and skills as a result of participating in the programme was substantial. For example, the majority of survey respondents somewhat agreed or strongly agreed that they felt more confident finding and reviewing the literature (24 of 29, 82.8%), disseminating their work (22 of 29, 75.9%), challenging practice using the evidence base (21 of 29, 72.4%) and supporting others to develop research skills (21 of 29, 72.4%). The majority also somewhat agreed or strongly agreed that they had a greater understanding of research design, methods and data analysis (26 of 29, 89.7%), the research process (25 of 29, 86.2%) and patient and public involvement in research (20 of 29, 69%). (See Table 4).

**Table 4:** Acquisition of specific research knowledge and skills as a result of participating in the iCAhRE programme

	Total sample	Strongly disagree, *n* (%)	Somewhat disagree, *n* (%)	Neither agree nor disagree, *n* (%)	Somewhat agree, *n* (%)	Strongly agree, *n* (%)
I have more confidence in finding relevant literature and critically reviewing it	29	2 (6.9)	0	3 (10.3)	7 (24.1)	17 (58.6)
I feel more confident to disseminate my work (e.g. presentations, posters, journal articles)	29	2 (6.9)	0	5 (17.2)	8 (27.6)	14 (48.3)
I have a greater understanding of research design, research methods and the analysis of data	29	2 (6.9)	0	1 (3.4)	13 (44.8)	13 (44.8)
I have a greater understanding of how to secure research funding	29	2 (6.9)	2 (6.9)	4 (13.8)	17 (58.6)	4 (13.8)
I have a greater understanding of the research processes e.g. ethics, research governance, etc.	29	2 (6.9)	0	2 (6.9)	13 (44.8)	12 (41.4)
I have a greater understanding of how to undertake patient and public involvement	29	2 (6.9)	0	7 (24.1)	13 (44.8)	7 (24.1)
I feel more confident to challenge practice using the evidence base	29	2 (6.9)	1 (3.4)	5 (17.2)	10 (34.5)	11 (37.9)
I have more confidence to help others to develop their research skills	29	1 (3.4)	2 (2.9)	5 (17.2)	8 (37.6)	13 (44.8)

Considering the programme overall, the majority of survey respondents (22 of 29, 75.9%) felt that it had increased their research knowledge and skills.

Within the open-ended survey responses and qualitative interviews, programme participants gave examples of the research skills or knowledge that iCAhRE had increased or improved. Several participants said that the programme had increased their knowledge of the research process:

*So*,* I know clinical research delivery … but I didn’t have a wide understanding of what research governance team do and what the set-up team do. … it sort of brought it all together … I had a better understanding of what everybody else was doing*,* you know*,* like governance and set up and things like that. (Interview participant 9).*

Programme participants also frequently said that the programme had increased their literature searching skills and their critical appraisal skills:

*The programme increased my level of understanding in literature reviewing and using research findings to inform practice. It has improved my critical and analytical skills (Open-text survey response).*

Writing skills was another key area where participants said that their skills had improved as illustrated by this example:

*Writing a research proposal. This wasn’t something I had done prior to INCA [currently iCAhRE Bronze]*,* but have been required to do a number of times since. Through participation in INCA I learnt how to go about developing a proposal*,* the format to use*,* and what needs to be included. (Open-text survey response).*

However, a small number of participants felt that the development of their research knowledge and skills was in its infancy and they needed more opportunities to develop these skills and to put them into practice.

The majority of survey respondents (18 of 29, 62%) felt that, overall, the programme had increased their confidence in research. The increase in participants’ confidence was a significant theme within the qualitative interviews over and above that indicated by the survey responses. All but one of the interviewees said that their confidence had increased as a result of the programme. They described how the programme had increased their confidence to undertake specific activities such as writing, presenting or undertaking research:

*Yes*,* I think it certainly gave me the idea that research is something that I could do. I knew I didn’t know everything. Nobody knows everything*,* do they? But it had given me enough understanding and confidence that I could go out and make a start with a little project*,* and that I could talk to people that I had some contacts within the hospital that were interested in research. (Interview participant 4).*

For some interviewees, the programme had increased their confidence to pursue a clinical academic career:

*[Aspects of the iCAhRE programme] were the really critical things to boost my confidence as a nurse working in a clinical area where*,* personally for me*,* I wasn’t around other researchers*,* a lot of my colleagues were clinical staff. So*,* to know that that [clinical academic training] pathway was out there*,* and to feel like I have the skills*,* feel like I have the knowledge and the confidence to do it. (Interview participant 2).*

### Applying new learning and impact on practice (Kirkpatrick level 3)

**Fig. 5:**
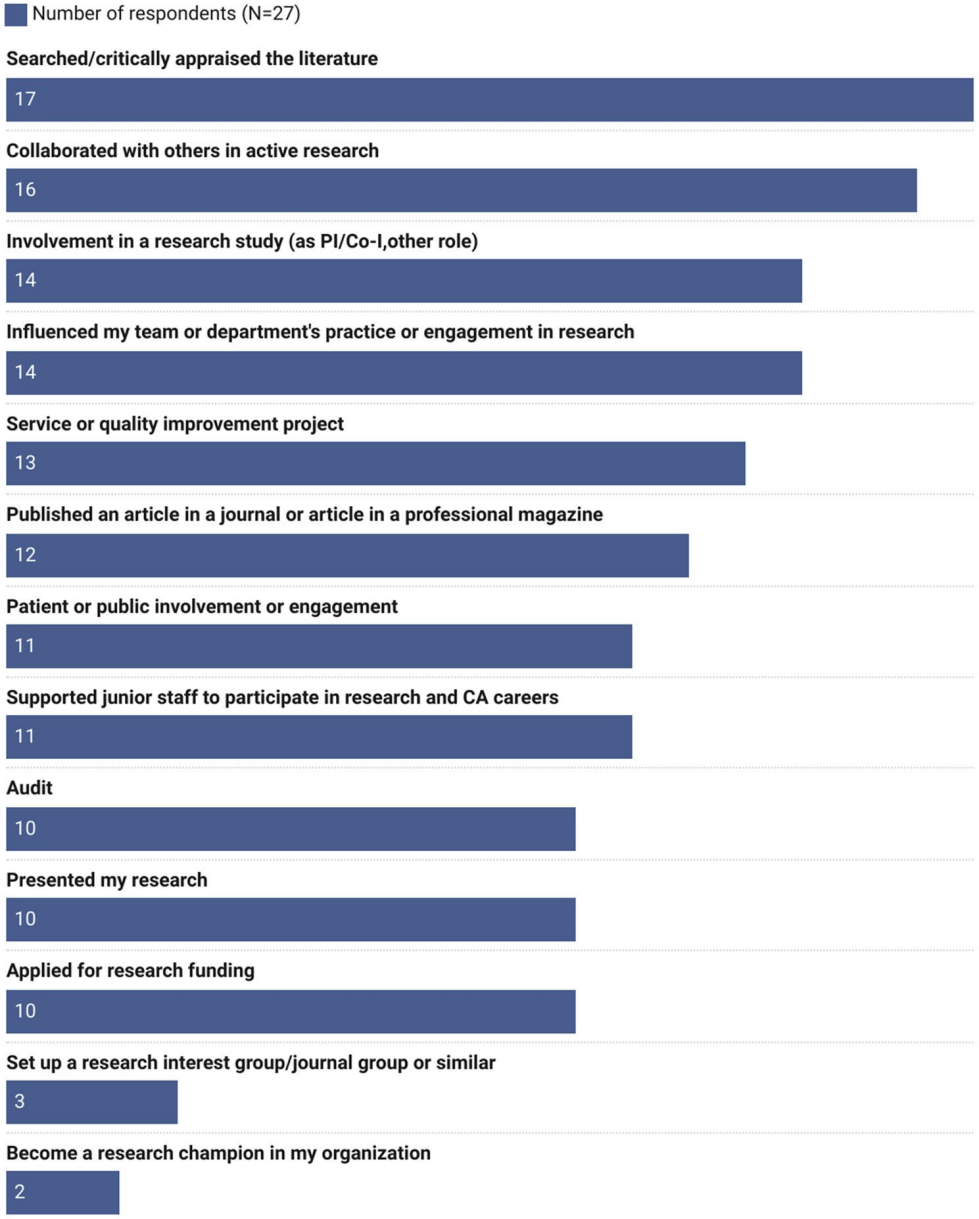
Activities undertaken during/following participation in the iCAhRE programme

The survey identified new activities initiated following involvement in the programme to enhance and deliver evidence-based practice and research literacy to improve quality of care. Figure 5 outlines the activities programme participants have undertaken in the workplace. Almost two-thirds (17 of 27, 63%) had searched or critically appraised the literature and over half (16 of 27, 59.3%) had collaborated with others in active research.

The interviews explored the initiation of research-related activities in more depth. Interviewees gave examples of service or quality improvement projects, setting up research interest groups, becoming involved in research projects and mentoring others:

*I have recently supported someone through the internship programme*,* so I’ve been their supervisor and increasingly*,* since I’ve started the PhD*,* I’ve started to mentor people who are doing … pre-doctoral applications. (Interview participant 10).*

Interviewees gave examples of how they had applied their learning from the iCAhRE programme. Half of interviewees had used their acquired literature searching skills to search and check the evidence base either to update guidance or to ascertain why a particular drug, test or practice was used. Other interviewees had used the writing skills they had gained from the programme to write journal articles and book chapters.

**Fig. 6:**
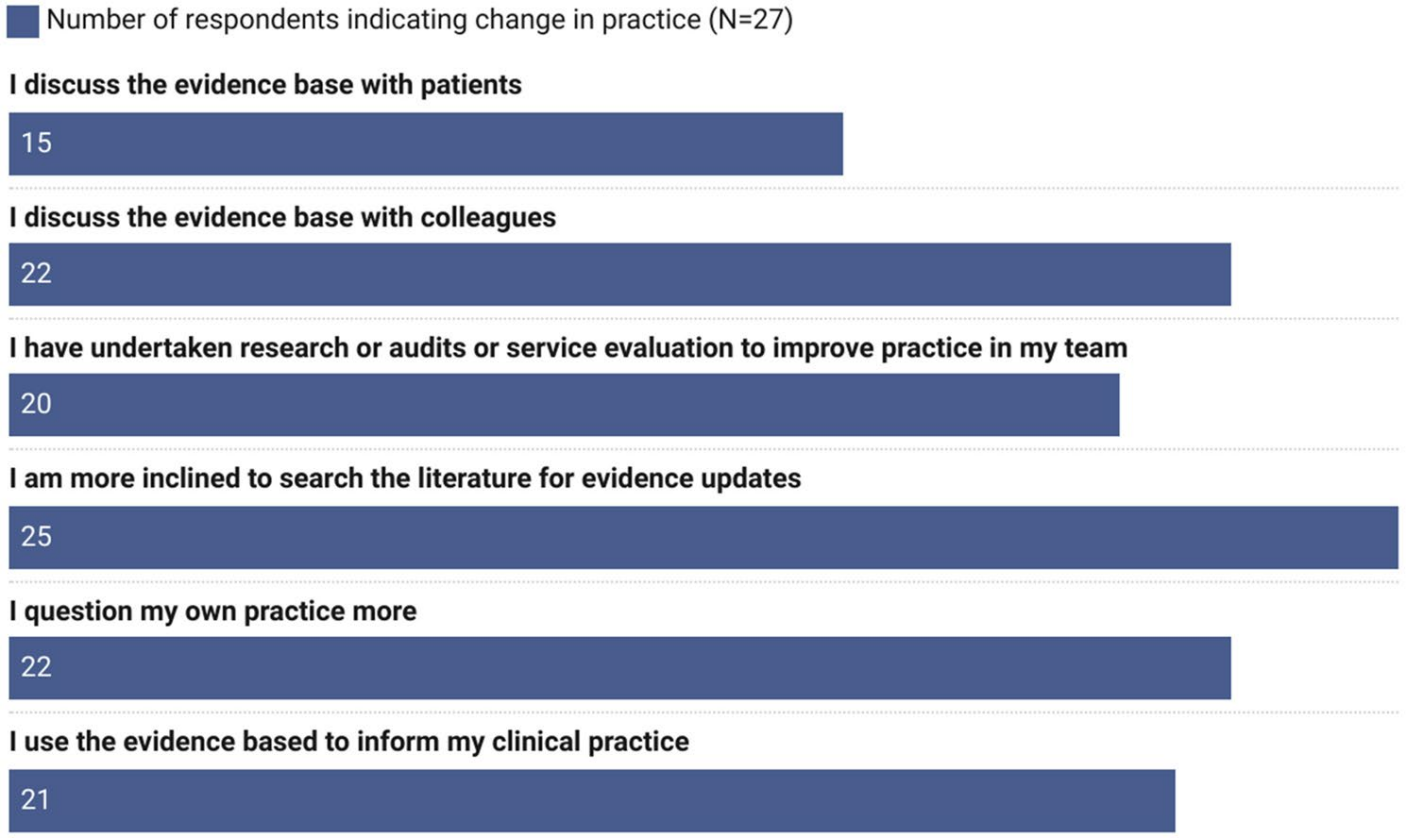
Changes in practice following participation in the iCAhRE programme

In addition, knowledge and skills developed during the programme provided perceived benefits for improving evidence-based practice which can lead to improvements in the quality of care delivered within services. Figure 6 shows that almost all survey respondents (25 of 27, 92.6%) reported they were more inclined to search the literature for evidence updates, 81.5% (22 of 27) of survey respondents reported they had discussed the evidence base with colleagues and 81.5% (22 of 27) reported they had questioned their own practice more.

The qualitative interviews illuminated examples of changes in practice. For example, interviewees described how they were more likely to discuss the evidence base with colleagues and how the evidence base can be used to inform clinical practice:

*Even when I’m discussing with someone [a colleague] and it comes up*,* “No*,* we used to do this at our place”*,* I’ll say*,* “Okay*,* give me the reason why we’re doing that? What are the benefits? And where does it come from? So*,* give me that and then we will introduce in my department.” You don’t just introduce because someone else has done that. It has to be evidence-based. (Interview participant 1)*

Participants were not always supported to make changes to their practice. One interview participant described trying to change their practice by spending time explaining to patients the purpose of a particular questionnaire using the evidence base but was criticised by managers for taking too much time with patients.

**Fig. 7:**
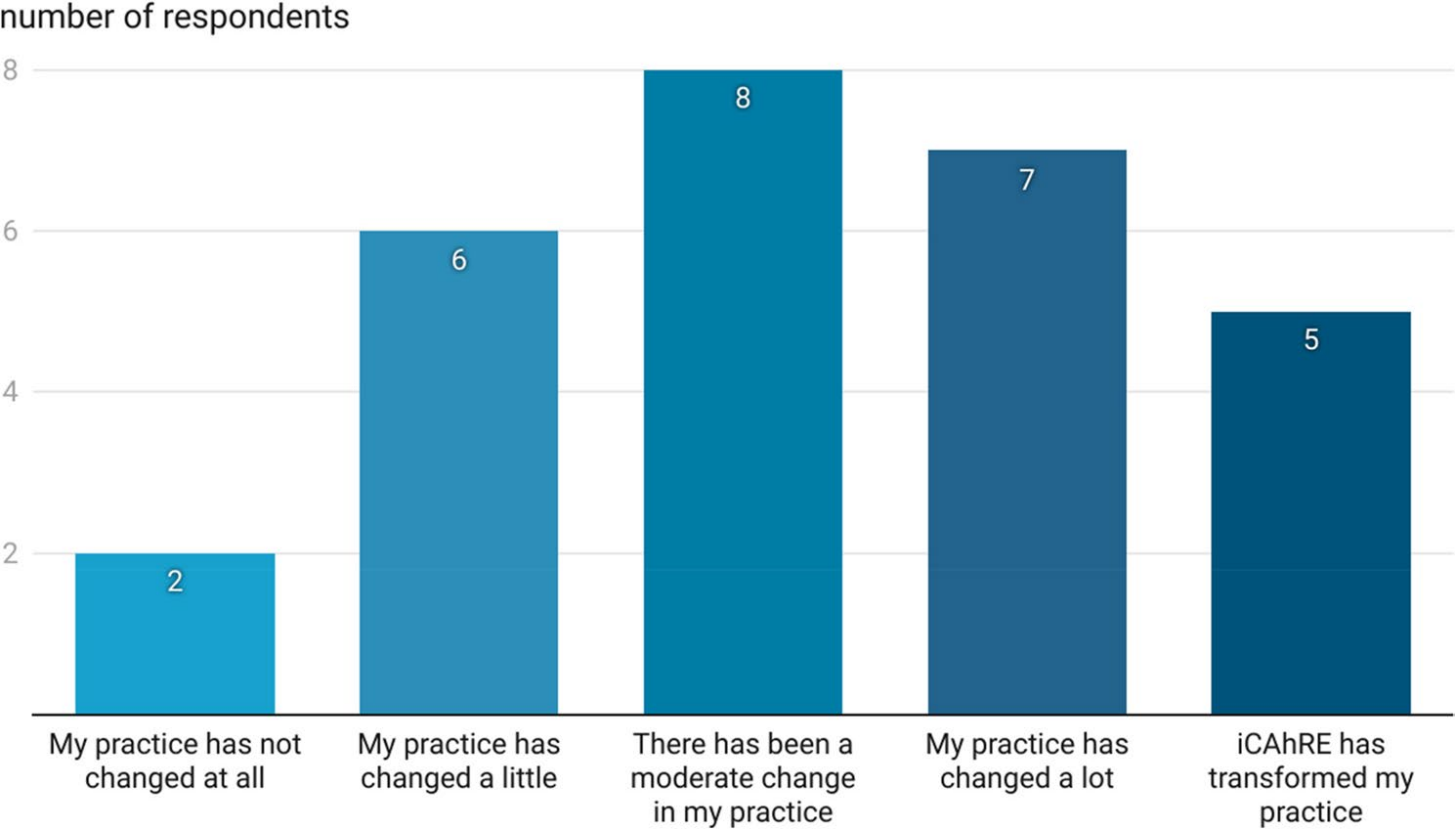
Extent of practice change since participating in the iCAhRE programme

Over two-fifths (12 of 28, 42.9%) of survey respondents reported that their practice had changed considerably since participating in the iCAhRE programme (see Fig. 7). Over a quarter of survey respondents (8 of 28, 28.6%) reported that there had been a moderate change in their practice and the same number (8 of 28, 28.6%) reported that their practice had not changed much or at all.

### Outcomes of the iCAhRE programme (Kirkpatrick level 4)

Role progression, having a role that included research, publications and research funding were used to evaluate whether the programme had achieved strategic goals and priorities. Survey respondents provided details of their role grade at the point they started the programme and their current role grade. Eleven respondents (37.9%) had progressed into a role that was at a higher grade (e.g. progressed from a Band 5 to a Band 6 role). Of these 11 respondents, 10 (90.9%) of them agreed that their participation in the programme had supported their career progression and one (9%) was unsure. For more than half of respondents (16, 55.2%) their role grading had not changed since they began the programme. However, six survey respondents had recently begun the programme (in July 2023) so limited time had elapsed for progression in their roles to occur. There was missing data for two respondents (6.9%).

For almost 60% of respondents (17, 58.6%), research was part of their current role. However, there were mixed views about the extent to which opportunities offered through the programme were crucial to securing a role that involves research. 40% of respondents (7, 41.1%) agreed that iCAhRE was important in helping them secure a role that involved research, but over a fifth (4, 23.5%) felt strongly that the programme did not help them secure a role that involved research. A further six respondents (35.3%) neither agreed nor disagreed that the programme helped them secure a role that involved research.

**Fig. 8:**
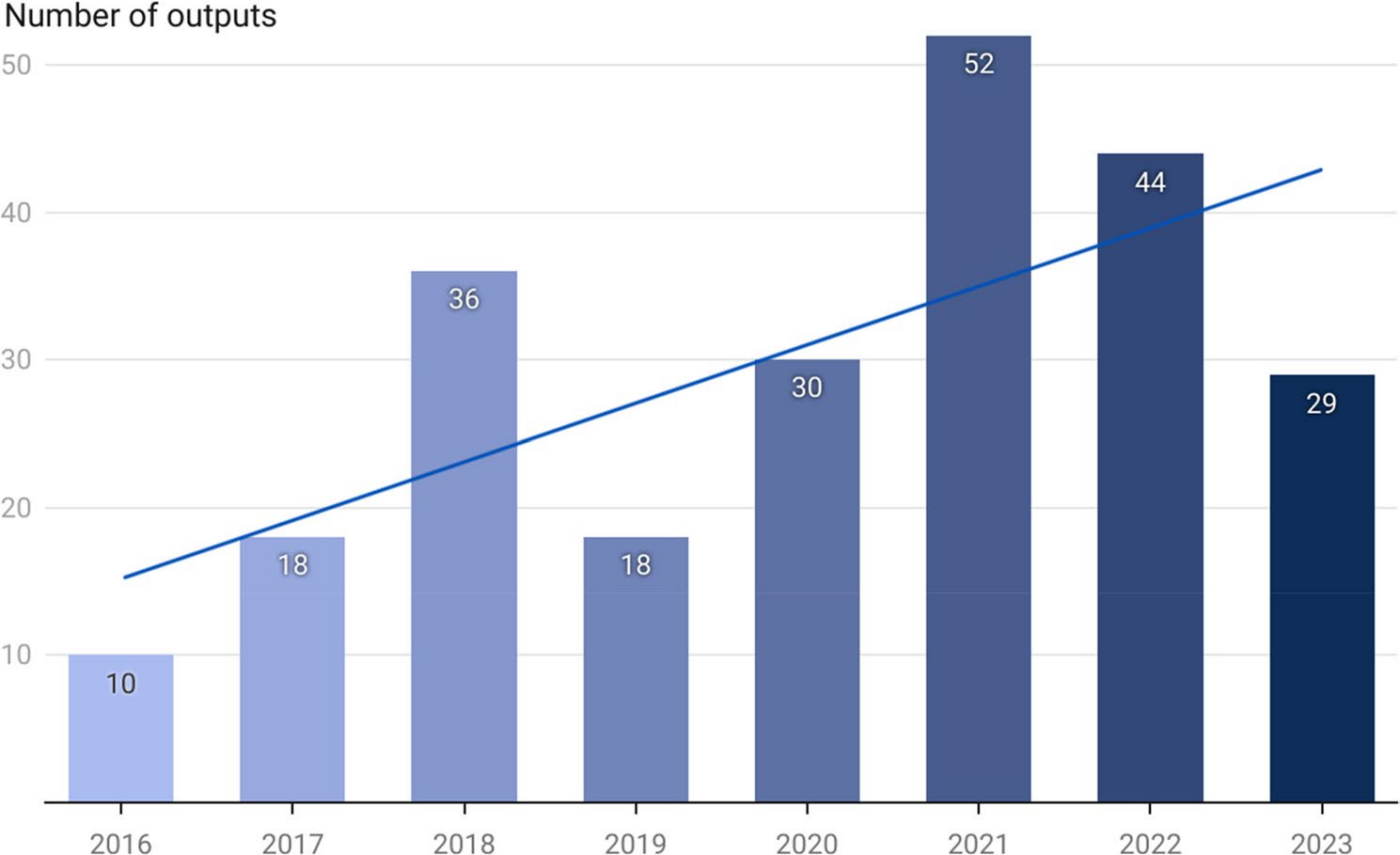
iCAhRE programme publications between 2016 and 2023 (total 237 publications)

The number of publications from programme participants increased gradually since the inception of the programme (see Fig. 8). Over 230 publications were produced between 2016 and 2023. Thirty-six programme participants published in this time period achieving a mean of 6.47 publications each. Not unexpectedly, Gold/Gold+ participants were more likely to have published, however just over a third (13, 36%) of the 36 programme participants who had published had completed the Bronze level of the programme only.

Between 2018 and 2023 (available range of data) 24 iCAhRE participants were awarded 40 research grants totalling £3.2 million (an average of just over £500,000 awarded per year). The mean award per participant was just over £133,000 (range: £340 to £1.2 million). These funding awards included 20 fellowships or other development awards.

### Enablers and barriers to progressing clinical academic careers

For many programme participants, the iCAhRE programme was an important enabler for clinical academic career progression. Several programme participants described how significant the programme had been to their careers:

*Looking back*,* I think iCAhRE has been really transformational for me*,* and I wouldn’t be at the stage where I am now without embarking*,* or without approaching R&D at that time about the iCAhRE programme. Clinically it has been a little bit up and down during the pandemic … But the research and iCAhRE programme actually kept me going and kept my resilience alive*,* because it was a time when I was quite frustrated with clinical roles. I was deployed in during the pandemic three times in critical care*,* and research was my only aspiration not to give up on the NHS and I kept my aspirations alive*,* it helped me to upskill my research skills and helped me to transform. (Interview participant 7).*

However, programme participants reported that they were often unsupported by managers and colleagues to pursue research and clinical academic training, research workshops and research funded by training and development awards. Substantial challenges, including national and more local hospital pressures, were reported in relation to support from managers who could block access to opportunities, did not understand or value research or who expressed support, but this support did not materialise in practice.

Overall, iCAhRE participants really valued protected time to pursue clinical academic training and a clinical academic career. This was an important enabler and included, for example, being released to engage with the programme or being awarded funding that supported their research time. However, there were considerable challenges in facilitating this. For example, there were practical challenges of finding cover for clinical time:

*It’s definitely time being released … How do you practically do it? Even if you’ve got the money to pay for somebody a day a week*,* where are you going to get somebody for a day a week? Or how are you going to spread the workload out? (Interview participant 3).*

Interviewees also identified challenges in taking time away from clinical work to undertake longer periods of study:

*You’ve got a lot of issues around covering services and stuff like that*,* that are practical issues that are going to stop [managers] being supportive … “If you’re going to do a four year PhD*,* I can’t keep your post open for four years. What are you going to do when you come back?” (Interview participant 6).*

Another practical challenge for staff wishing to progress a clinical academic career was the possible financial impact. This could include clinical academic roles being at a lower pay banding than their current clinical role, moving to HEIs where pay scales can be lower, especially at senior levels, and concerns around retaining an NHS pension. These financial challenges were more significant for those who were closer to retirement.

Programme participants described a lack of clinical academic posts, particularly at more junior levels, and limited progression opportunities as a significant barrier:

*So*,* I think that what happens next [after a PhD] is probably the difficult question for non-medic clinical academics … My ideal would still be to be part clinical*,* part research and part leadership. I would like to create a role like that. I would like to stay at UHCW … but you’re relying on them to create those [opportunities] to some extent … I can see where I want to be*,* but I can’t necessarily see all the steps to get there. (Interview participant 10).*

In terms of increased knowledge and skills, iCAhRE participants wanted these attributes to be reflected in their banding, but often felt that this was not the case:

*We cannot expect clinical academic researchers to continue to be at the same band or pay rate*,* even if they have completed their PhD*,* or even if they’ve achieved an MRes*,* or even if they’ve shown some leadership nationally*,* or changed clinical practice. (Interview participant 7).*

Some interviewees also felt that lack of visibility and understanding of clinical academic career pathways was a barrier to pursuing a clinical academic career because staff may not be aware that a clinical academic career is a possibility. This is compounded by a lack of senior clinical academics in certain professions.

## Discussion

The existence of the iCAhRE programme since 2016 has provided a unique opportunity to evaluate the longer-term impacts of a facilitated research career pathway which is frequently lacking in the current evidence base [25]. The mixed methods approach generated key data against all four levels of Kirkpatrick’s evaluation model (reaction, learning, behaviour and results) as well as assessing the reach of the programme. Quantitative data demonstrates that the programme is enabling staff to progress their clinical academic careers by supporting staff to start at an introductory level (Bronze) before moving to other levels and also by supporting staff to access levels of iCAhRE commensurate with their previous level of training and experience (entering the programme at Silver or Gold/Gold+). However, there was limited progression between the levels of the programme suggesting the presence of barriers to career development which counteract the opportunities offered by the pathway. These barriers were illuminated in the interviews and survey free text comments. Nurses and midwives were underrepresented and allied health professionals (AHPs) were overrepresented when Bronze/Silver/Gold/Gold+ data and organisation level data were compared. This warrants further exploration within the programme as a whole to highlight any issues with diversity and inclusion.

Responses to the programme were highly positive. Participants accessed a wide range of types of support within the programme and reported high levels of satisfaction. Participants were complimentary about the staff involved in the programme, aligning with previous research [22]. The key area where staff had significant concerns was around the NHS Trust not having enough research opportunities for staff.

Programme participants agreed that they had increased their research knowledge and skills overall through participation in the programme. Previous research has already identified increased confidence in research amongst participants of clinical academic training programmes [20, 22] and in this study the increased confidence of participants as a result of the programme was particularly notable. Programme participants had initiated new research-related activities following involvement in the programme. The programme also had an impact on practice; many participants reported a considerable or moderate change to their practice. This finding was consistent with evaluations of other similar programmes [20, 22].

Metrics of success for the programme included Agenda for Change (AfC) band progression, being in a role that included research, publications and funding. Just over a third of survey participants had progressed to roles with a higher banding compared to when they started the programme, a slightly higher proportion than reported by Hiley and colleagues [20] in their evaluation although different timescales are likely to account for this. In our study, most participants felt that their participation in the programme had supported their career progression. Over 230 publications had been produced at the time of evaluation by iCAhRE participants and they had been awarded research grants totalling £3.2 million. These metrics of success are evidence of the interconnection between investment in the programme and staff who participate, deliver and lead it and programme outcomes. Economic evaluation of the costs and benefits of investments in enhancing research capability and capacity would be an important area for future research.

There are strong arguments for local responsiveness alongside national initiatives to increase opportunities for research capability and capacity [29] and the iCAhRE programme is an example of such a local approach. Like other similar approaches [20, 22, 23] the programme has made inroads in addressing the capacity gap. However, support from the programme has been insufficient to address some of the more deep-seated challenges to pursing a clinical academic career. Key elements of the programme and findings from the evaluation are now explored in relation to the three themes (‘supporting healthcare professionals in research^2^’, ‘working together’ and ‘valuing research for excellence’) of Matus and colleagues’ integrated framework for research capacity building [30] to illuminate how the programme and its infrastructure have supported clinical academic career development and where challenges still remain.

### Supporting healthcare professionals in research

The iCAhRE programme, like other locally based programmes in other areas of England [19, 21], offers a wide range of opportunities for NMAHP+s that support their engagement in research and research training. For example, the programme, particularly the Bronze module, offers relevant education and training that relates to aspects of the research process such as writing grant and ethics applications, opportunities to learn and apply skills in practice, mentoring and coaching from more experienced researchers, support to apply for research funding and most significantly support to undertake formal post-graduate study including higher degrees by research.

As well as developing the content of the programme, the iCAhRE team has created a culture within the NHS Trust to support NMAHP+s to undertake the programme. This has included Trust-wide advertising of the programme, communication and celebration of achievements. Protected time to undertake research and training was an important enabler and was valued by programme participants [10, 11], but they identified considerable challenges in achieving this. Previous studies have also identified lack of protected time as a barrier to career progression [10–12]. Matus and colleagues [30] highlight the importance of career opportunities, research career pathways and financial incentives, but limited opportunities for progression and a lack of clinical academic posts were significant themes in our study, echoing the wider literature [15, 31–33]. This is an area of ongoing strategic focus [16, 18, 34].

### Working together

Partnerships and collaborative working needed to be developed at the start of the programme but are considered crucial elements of capacity building and facilitators for clinical academic career development [14, 23, 35]. A key foundation of the iCAhRE programme is the long-term strategic partnership between the NHS Trust and Coventry University. The programme was developed and continues to be refined collaboratively between these two organisations. Key members of the programme’s leadership team, a clinically focused university professor and the NHS Trust Head of R&D, have remained consistent since the inception of the programme and this has supported the focus and drive to achieve the programme’s delivery outcomes. This long-term strategic partnership has been critical to achieving the programme’s impact.

### Valuing research for excellence

Demonstrating visible support for research has been critical for the iCAhRE programme. This has been achieved through strong and consistent leadership of the programme and consistent endeavours by R&D to raise the profile of research in the NHS Trust such as local, regional, national and international presentations, communication on the website, Trust-wide research events, and the appointment of iCAhRE participants to senior roles in HEIs and research funding bodies. Research is prioritised as part of the NHS Trust’s core business as it is one of the five purposes of the organisation and included in the organisation’s values and Organisational Strategy. Previous research suggests that strong and visible leadership and management buy-in is a key element within other local approaches [19, 21]. However, senior leadership support did not necessarily translate into wider support within the healthcare organisation. A lack of support from managers was an important theme in our research reflecting findings from previous studies that suggest that unsupportive managers are a key barrier, potentially because they do not appreciate and understand the benefits of NMAHP+-led research and may be under pressure to prioritise clinical work due to high clinical demand [13–15]. Visible role models can be an enabler for successful clinical academic career pathways, but these are not present for all professional groups which can act as a barrier reflecting existing research findings [15, 32, 36].

### Strengths and limitations

A particular strength of this study is its mixed methods design which has enabled generation of a wide range of quantitative and qualitative data addressing the four levels of Kirkpatrick’s framework. The existence of the programme since 2016 enabled assessment of its impact over the longer term. The response rate to the survey was good. However, some staff had left the NHS Trust and were uncontactable so the survey did not reach all programme participants. Data was not available on age, ethnicity, part/full-time working, disability and caring responsibilities for the whole iCAhRE participant population preventing a fuller description of the group and a fuller examination of reach. Findings report key components of the programme that were valued and areas of concern; this information can be used to improve future training in this setting and others whilst recognising that was a single facilitated research career pathway provided in one NHS Trust and there may be a limit to the generalisability of the findings to other settings which will have their own local contextual factors influencing responses to the programme.

## Conclusion

This evaluation covers the 8-year period that the iCAhRE programme has been in existence. This NHS Trust-supported programme provides high quality training and development opportunities and participants indicated high levels of satisfaction. The programme appears to have lasting effects, increasing research knowledge and skills, informing and changing participants’ clinical practice, building confidence, increasing research-related activities undertaken and career progression. This has been achieved through a programme that offers a wide range of opportunities for NMAHP+s that support their engagement in research and research training. Also key to the achievement of the programme’s impact has been the long-term strategic partnership between the NHS Trust and Coventry University and the visible organisational support for research within the organisation. This demonstrates the importance of strong collaboration between NHS and academic institutions and how critical visible organisational support for research is. However, some of the more deep-seated challenges to pursing a clinical academic career persisted for programme participants. The findings suggest that there is substantial scope for greater provision of opportunities for progression and creation of clinical academic posts as well as addressing the issue of unsupportive managers and ensuring greater visibility of clinical academic NMAHP+ role models. Overall, the programme has the potential to be adopted and adapted elsewhere to nurture research-active environments and promote research capacity building.

## Supplementary Information

Supplementary Material 1

Supplementary Material 2

## Data Availability

The datasets used and/or analysed during the current study are available from the corresponding author on reasonable request.

## Abbreviations

AHP: Allied Health Professional
HEE: Higher Education England
HEI: Higher Education Institute
iCAhRE: Interdisciplinary Clinical Academic Health Research Excellence facilitated research career pathway
NHS: National Health Service
NIHR: National Institute for Health and Care Research
NMAHP+s: Nurses, Midwives, Allied Health Professionals and Healthcare Scientists
R&D: Research and Development
UHCW: University Hospitals Coventry & Warwickshire NHS Trust

## Footnotes

1 In the context of the iCAhRE programme, capability refers to the skills, knowledge, and expertise of the participants.

2 We have substituted the term ‘healthcare professionals’ for the original term ‘clinicians’ used by Matus and colleagues.
