# Supplementary material for: A mixed methods evaluation of a facilitated research career pathway for nurses, midwives, allied health professionals and healthcare scientists working in the NHS
Source: BMC Med Educ. 2025 Oct 17;25:1438. doi: 10.1186/s12909-025-07982-2 (PMC12532386; doi:10.1186/s12909-025-07982-2)
Supplement: Supplementary file 1 — Supplementary Material 1 [file 12909_2025_7982_MOESM1_ESM.docx]

**iCAhRE Evaluation Survey**

**Section 1: Tell us about your participation in iCAhRE**

Thank you very much in advance for your help with this research evaluating the iCAhRE programme. First, we would like to know about your participation in the iCAhRE programme (interdisciplinary Clinical Academic health Research Excellence). iCAhRE was previously known as the Interdisciplinary Non-medical Clinical Academic (INCA) Research Programme so when we refer to iCAhRE we also mean INCA too.

**How did you find out about the iCAhRE (formerly INCA) programme?**

- Staff newsletter
- From my manager
- From my colleague
- From the Research and Development (R&D) team
- From the Centre for Care Excellence team
- Other (please specify) __________________________________________________

**What level(s) of the iCAhRE programme have you completed? (Select all that apply)**

- **iCAhRE Bronze module (formerly INCA Bronze) with Coventry University.**  Research-based learning provided by Coventry University, with some staff undertaking a Masters level research module. Also includes real life understanding of clinical research using a portfolio. Stage is ‘introductory’ to prepare clinical non-medical professionals to apply for a Masters in research or similar programme.
- **CAIP (Clinical Academic Internship Programme) Birmingham Health Partners** Research-based learning provided by Birmingham University, with some staff undertaking a Masters level research module. Stage is ‘introductory’ to prepare clinical non-medical professionals to apply for a Masters in research or similar programme.
- **iCAhRE Silver – pre MRes or MRes or similar** Includes any support to prepare staff to apply for a Masters or support post-Masters to apply for other research funding. Support might include help with applications, advice on interviews etc. Masters may be delivered in Coventry University or by other Higher Education Institution providers (such as Birmingham or Lincoln Universities).
- **iCAhRE Gold – PhD** Includes any support to apply for a PhD or PhD funding or support to complete a PhD from the iCAhRE programme.
- **iCAhRE Gold+ post doctoral support** Includes any support to apply for research or other funding post doctorate.

Display this question:

If What level(s) of the iCAhRE programme have you completed? (Select all that apply) = <strong>iCAhRE Bronze module (formerly INCA Bronze) with Coventry University. </strong> Research-based learning provided by Coventry University, with some staff undertaking a Masters level research module. Also includes real life understanding of clinical research using a portfolio. Stage is ‘introductory’ to prepare clinical non-medical professionals to apply for a Masters in research or similar programme.

**If you took part in iCAhRE Bronze (formerly INCA Bronze), when did you start?**

- May 2016
- November 2016
- January 2018
- January 2020
- July 2023
- Other (please specify) __________________________________________________
- Unsure/can't remember

**What support have you received from the iCAhRE (formerly INCA) team (including R&D or Centre for Care Excellence staff) before or after applying for the programme? (Please select all that apply)**

- Applications review and feedback
- Signposting to the iCAhRE or INCA programme or other funding opportunity
- Interview preparation/mock interviews
- Portfolio development, CV review and development
- Skills development workshops e.g. writing workshops, statistics support
- Signposting to other relevant resources/people (e.g. wider R&D teams, Public & Patient Involvement & Engagement (PPIE), statistician, Research Design Service)
- Signposting to various opportunities (grants, events, workshops, fellowships)
- Mentoring or leadership support
- Other (please specify) __________________________________________________

| Page Break |  |
| --- | --- |

**Section 2: What did you gain from the iCAhRE programme?**

Now that you have told us about your participation in the iCAhRE programme, we would like to know what you gained from iCAhRE. Specifically, we would like to know how iCAhRE increased your research knowledge and skills.

**As a result of participating in iCAhRE (formerly INCA):**

|  | Strongly disagree | Somewhat disagree | Neither agree nor disagree | Somewhat agree | Strongly agree |
| --- | --- | --- | --- | --- | --- |
| I have more confidence in finding relevant literature and critically reviewing it |  |  |  |  |  |
| I feel more confident to disseminate my work (e.g. presentations, posters, journal articles) |  |  |  |  |  |
| I have a greater understanding of research design, research methods and the analysis of data |  |  |  |  |  |
| I have a greater understanding of how to secure research funding |  |  |  |  |  |
| I have a greater understanding of the research processes e.g. ethics, research governance, etc. |  |  |  |  |  |
| I have a greater understanding of how to undertake patient and public involvement |  |  |  |  |  |
| I feel more confident to challenge practice using the evidence base |  |  |  |  |  |
| I have more confidence to help others to develop their research skills |  |  |  |  |  |

**Please indicate how much you agree or disagree with the following: iCAhRE (formerly INCA) increased my research knowledge and skills**

- Strongly disagree
- Somewhat disagree
- Neither agree nor disagree
- Somewhat agree
- Strongly agree

Display this question:

If Please indicate how much you agree or disagree with the following: iCAhRE (formerly INCA) increas... = Somewhat agree

Or Please indicate how much you agree or disagree with the following: iCAhRE (formerly INCA) increas... = Strongly agree

**Please explain or give an example of the research skills or knowledge that iCAhRE (formerly INCA) increased or improved**

________________________________________________________________

________________________________________________________________

________________________________________________________________

________________________________________________________________

________________________________________________________________

|  |  |
| --- | --- |

**Section 3: Impact of the iCAhRE programme**

We are interested in whether participating iCAhRE has prompted you to undertake particular research-focused activities, whether it has influenced your practice and helped with your career.

**What activities have you undertaken during/following participation in the iCAhRE (formerly INCA) programme? (Select all that apply)**

- Searched/critically appraised the literature
- Audit
- Service improvement or quality improvement project
- Involvement in a research study (as Principal Investigator (PI)/Co-Investigator/other role)
- Collaborated with others in active research e.g. research groups
- Published an article in a journal or article in a professional magazine
- Presented my research (e.g. oral presentation, poster, at a conference)
- Patient or public involvement and / or engagement
- Influenced my team / department's practice or their engagement in research
- Supported junior staff to participate in research and clinical academic careers
- Set up a research interest group / journal group or similar
- Become a research champion in my organization
- Applied for research funding (whether successful or not)

**How much do you agree that iCAhRE (formerly INCA) has increased your confidence in research?**

- Strongly disagree
- Somewhat disagree
- Neither agree nor disagree
- Somewhat agree
- Strongly agree

**How has your practice changed during or since participating in iCAhRE (formerly INCA)?**

|  | Yes | No | Not applicable (e.g. I no longer work in clinical practice) |
| --- | --- | --- | --- |
| I discuss the evidence base with patients |  |  |  |
| I discuss the evidence base with colleagues |  |  |  |
| I have undertaken research or audits or service evaluation to improve practice in my team |  |  |  |
| I am more inclined to search the literature for evidence updates |  |  |  |
| I question my own practice more |  |  |  |
| I use the evidence based to inform my clinical practice |  |  |  |
| Other (please specify) |  |  |  |

**Overall, to what extent has your practice changed since participating in iCAhRE (formerly INCA)?**

|  | My practice has not changed at all | My practice has changed a little | There has been a moderate change in my practice | My practice has changed a lot | iCAhRE has transformed my practice |
| --- | --- | --- | --- | --- | --- |
| Change in practice |  |  |  |  |  |

Display this question:

If Overall, to what extent has your practice changed since participating in iCAhRE (formerly INCA)? = My practice has not changed at all

Or Overall, to what extent has your practice changed since participating in iCAhRE (formerly INCA)? = My practice has changed a little

**You say that your practice has not really changed since participating in iCAhRE (formerly INCA), why is that? (Select all that apply)**

- Not enough time
- No support from manager
- No support from team
- Realised research is not for me
- Not confident enough
- Other (please specify __________________________________________________

**In your own words, how has the research training you have received through iCAhRE helped you in your career and/or clinical practice?**

________________________________________________________________

________________________________________________________________

________________________________________________________________

________________________________________________________________

________________________________________________________________

**In your own words, how has the research training you have received through iCAhRE improved patient care?**

________________________________________________________________

________________________________________________________________

________________________________________________________________

________________________________________________________________

________________________________________________________________

**Section 4: Achievements arising from participation in iCAhRE**

So that we can capture the continued impact of iCAhRE, this section of the survey asks about your achievements arising from your participation in iCAhRE.

**Please could you tell us your job title when you started iCAhRE (formerly INCA)?**

________________________________________________________________

________________________________________________________________

________________________________________________________________

________________________________________________________________

________________________________________________________________

|  |  |
| --- | --- |

**What grade was that role?**

- Band 5
- Band 6
- Band 7
- Band 8A
- Band 8B
- Band 8C
- Band 8D
- Band 9
- Other (e.g. University grading)

**What is your job title currently?**

________________________________________________________________

________________________________________________________________

________________________________________________________________

________________________________________________________________

________________________________________________________________

|  |  |
| --- | --- |

**What grade is your current role?**

- Band 5
- Band 6
- Band 7
- Band 8A
- Band 8B
- Band 8C
- Band 8D
- Band 9
- Other (e.g. University grading) similar grade to before
- Other (e.g. University grading) higher grade than before

Display this question:

If Bandnow > ${q://QID36/SelectedChoicesRecode}

**Do you think that your participation in the iCAhRE (formerly INCA) programme supported you in your career progression?**

- Yes
- No
- Unsure

**Please provide details of any publications, conference presentations/posters you have achieved since participating in iCAhRE (formerly INCA) (e.g. number of journal articles, number of articles in professional magazines, number of conference presentations/posters)?**

- Journal articles/papers (specify number) __________________________________________________
- Article in a professional magazine (specify number) __________________________________________________
- Conference presentations/posters (specify number __________________________________________________
- Other, please specify what and number __________________________________________________

**Have you applied for any research training awards (e.g. NIHR/HEE internships, PCAF, DCAF, ACAF) since participating in iCAhRE (formerly INCA)?**

- Yes
- No

Display this question:

If Have you applied for any research training awards (e.g. NIHR/HEE internships, PCAF, DCAF, ACAF) s... = Yes

**If yes, please provide details including scheme applied for, funder and whether this was successful.**

________________________________________________________________

________________________________________________________________

________________________________________________________________

________________________________________________________________

________________________________________________________________

**Have you applied for any research funding/grants since participating in iCAhRE (formerly INCA) (including successful and unsuccessful)?**

- Yes
- No

Display this question:

If Have you applied for any research funding/grants since participating in iCAhRE (formerly INCA) (i... = Yes

**If yes, please provide details including amount applied for, funder and whether this was successful.**

________________________________________________________________

________________________________________________________________

________________________________________________________________

________________________________________________________________

________________________________________________________________

**Is research part of your current role?**

- Yes
- No

Display this question:

If Is research part of your current role? = Yes

**Please rate the degree to which you agree with this statement: Opportunities offered through the iCAhRE (formerly INCA) programme were crucial to me securing a role that involves research.**

- Strongly disagree
- Somewhat disagree
- Neither agree nor disagree
- Somewhat agree
- Strongly agree

|  |  |
| --- | --- |

**Section 5: Feedback on the iCAhRE programme**

Thank for your responses so far. This final section of the survey asks what you think about the iCAhRE programme e.g. what you like about it and what could be improved.

**Please rate the degree to which you agree with the statements below:**

|  | Strongly disagree | Somewhat disagree | Neither agree or disagree | Somewhat agree | Strongly agree | Not applicable |
| --- | --- | --- | --- | --- | --- | --- |
| I am clear about what the iCAhRE programme, purpose and strategy is |  |  |  |  |  |  |
| I enjoyed the iCAhRE programme |  |  |  |  |  |  |
| iCAhRE considered my individual journey |  |  |  |  |  |  |
| Because of the iCAhRE team, I am/I was aware of many research related opportunities such as grants, events and fellowships |  |  |  |  |  |  |
| Managers at UHCW support research |  |  |  |  |  |  |
| UHCW has enough research development opportunities for staff |  |  |  |  |  |  |
| iCAhRE is essential for developing a research career (it not be done without it) |  |  |  |  |  |  |
| I would encourage my colleagues to seek support from iCAhRE as a first option |  |  |  |  |  |  |

**Overall from 1-10 please rate how satisfied are you with the support and opportunities you have been offered through the iCAhRE (formerly INCA) programme**

- 1 - not at all satisfied
- 2
- 3
- 4
- 5
- 6
- 7
- 8
- 9
- 10 - extremely satisfied

**Please add any additional information to explain why you have given this rating? (optional)**

________________________________________________________________

________________________________________________________________

________________________________________________________________

________________________________________________________________

________________________________________________________________

**To what extent would you recommend the iCAhRE (formerly) programme to others?**

- 1 - would NOT recommend
- 2
- 3
- 4
- 5
- 6
- 7
- 8
- 9
- 10 - would definitely recommend

**Please add any additional information to explain why you have given this rating? (optional)**

________________________________________________________________

________________________________________________________________

________________________________________________________________

________________________________________________________________

________________________________________________________________

**What was the most beneficial aspect of iCAhRE (formerly INCA)?**

________________________________________________________________

________________________________________________________________

________________________________________________________________

________________________________________________________________

________________________________________________________________

**What recommendations do you have for developing and improving the iCAhRE programme in the future?**

________________________________________________________________

________________________________________________________________

________________________________________________________________

________________________________________________________________

________________________________________________________________

**Finally, are there any other comments you would like to add about the iCAhRE (formerly INCA) programme or any other aspect of Research & Development at UHCW NHS Trust?**

________________________________________________________________

________________________________________________________________

________________________________________________________________

________________________________________________________________

________________________________________________________________

|  |  |
| --- | --- |

**Section 6: Your role**

We would like to know a bit about your current role to help us understand more about the roles that people who have taken part in/are taking part in iCAhRE do.

**Are you currently a University Hospitals Coventry and Warwickshire (UHCW) NHS Trust employee?**

- Yes - Still at UHCW NHS Trust
- No - Moved to another NHS Trust
- No - Moved to a regional or national role (e.g. NIHR, NHS England)
- No - Moved to a University / other academic institution
- No - Moved to the private sector
- No - Moved to a charity
- No – self-employed
- No – caring responsibilities
- No – retired
- Doing something else (please specify) __________________________________________________

Display this question:

If Are you currently a University Hospitals Coventry and Warwickshire (UHCW) NHS Trust employee? = No - Moved to another NHS Trust

And Are you currently a University Hospitals Coventry and Warwickshire (UHCW) NHS Trust employee? = No - Moved to a regional or national role (e.g. NIHR, NHS England)

And Are you currently a University Hospitals Coventry and Warwickshire (UHCW) NHS Trust employee? = No - Moved to a University / other academic institution

And Are you currently a University Hospitals Coventry and Warwickshire (UHCW) NHS Trust employee? = No - Moved to the private sector

And Are you currently a University Hospitals Coventry and Warwickshire (UHCW) NHS Trust employee? = No - Moved to a charity

And Are you currently a University Hospitals Coventry and Warwickshire (UHCW) NHS Trust employee? = No – self-employed

And Are you currently a University Hospitals Coventry and Warwickshire (UHCW) NHS Trust employee? = No – caring responsibilities

And Are you currently a University Hospitals Coventry and Warwickshire (UHCW) NHS Trust employee? = No – retired

And Are you currently a University Hospitals Coventry and Warwickshire (UHCW) NHS Trust employee? = Doing something else (please specify)

**You say that you have left UHCW NHS Trust, why was that?**

- Promotion
- Moved house / area
- No change in grade but more opportunities for development
- My research ideas were not supported
- Not enough opportunities to pursue a research career at UHCW NHS Trust
- Successful Fellowship or grant application
- Better work/life balance
- Other (please specify) __________________________________________________
- Prefer not to say

**What is your professional group?**

- Additional Professional Scientific and Technical
- Additional clinical services
- Admin & Clerical
- Allied Health Professionals
- Estates and Ancillary
- Healthcare Scientists
- Medical and Dental
- Nursing and Midwifery (Registered)
- Other (please specify) __________________________________________________
- Prefer not to say

**What is your speciality group?**

- Core
- Emergency Medicine
- Medicine
- Trauma & Neuro
- Surgery
- Women and Children's
- Clinical Diagnostics
- Clinical Support Services
- Other (please specify) __________________________________________________
- Not applicable

**If you are a qualified/registered healthcare or other professional, how many years post qualification/registration experience do you have?**

- 0-5 years
- 6-10 years
- 11-15 years
- 16-20 years
- 21-25 years
- 30+ years
- Prefer not to say
- Not applicable

**If you are a qualified/registered healthcare or other professional, did you train internationally (e.g. train outside of the UK)?**

- Yes
- No
- Prefer not to say
- Not applicable

**In your current role, do you spend any time on research activity?**

- Yes
- No
- Prefer not to say

Display this question:

If In your current role, do you spend any time on research activity? = Yes

**Approximately how much time (percentage) do you spend on research activity in your current role?**

|  | 0 | 10 | 20 | 30 | 40 | 50 | 60 | 70 | 80 | 90 | 100 |
| --- | --- | --- | --- | --- | --- | --- | --- | --- | --- | --- | --- |

| How much time (percentage) spent on research activity | 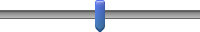 |
| --- | --- |

**I am employed:**

- Full-time
- Part-time
- Prefer not to say
- Not applicable

|  |  |
| --- | --- |

**Section 7: Some details about you**

Finally, we are committed to inclusive research and so would like you to provide some information to help us understand the responses to the survey. Please could you complete these questions that ask for a few details about you. They are optional to complete.

**What is your age group?**

- 21-30 years
- 31-40 years
- 41-50 years
- 51+ years
- Prefer not to say

**What best describes your gender:**

- Female
- Male
- Prefer to self-describe: __________________________________________________
- Prefer not to say

**Is your gender identity the same as the sex you were registered at birth?**

- Yes
- No
- Prefer not to say

**How would you describe your ethnic group? (Choose one option that best describes your ethnic group or background)**

- English, Welsh, Scottish, Northern Irish or British
- Irish
- Romany, Gypsy or Traveller
- Any other White background
- White and Black Caribbean
- White and Black African
- White and Asian
- Any other Mixed or Multiple ethnic background
- Indian
- Pakistani
- Bangladeshi
- Chinese
- Any other Asian background
- Caribbean
- African
- Any other Black, African or Caribbean background
- Arab
- Any other ethnic group
- Prefer not to say

**Do you have any long-standing illness, disability or infirmity? (Long-standing means anything that has troubled you over a period of time or that is likely to affect you over a period of time)?**

- Yes
- No
- Prefer not to say

**Do you have any parental/caring responsibilities?** (For example, do you have any children aged from 0-17 living at home with you, or who you have regular caring responsibilities for? Do you look after, or give any help or support to family members, friends, neighbours or others because of either: long term physical or mental ill health / disability, or problems related to old age?)

- Yes
- No
- Prefer not to say

|  |  |
| --- | --- |

**Section 7: Taking part in an interview**

We are also interested in speaking to people in more depth about their experiences of the iCAhRE programme. This could take place over the phone or Zoom/Microsoft Teams, and would last around 60 minutes. If you are interested, please click here to find out more.

**Would you be interested in taking part in an interview with a researcher from Coventry University?**

- Yes, I am interested in being interviewed
- No, I am not interested in being interviewed

Display this question:

If Would you be interested in taking part in an interview with a researcher from Coventry University? = Yes, I am interested in being interviewed

Please write your name and contact details below, and a researcher will contact you.

Instead, if you wish to submit your survey responses anonymously, you can email Eleanor on eleanor.lutman-white@coventry.ac.uk for more information about taking part in an interview.

Display this question:

If Would you be interested in taking part in an interview with a researcher from Coventry University? = Yes, I am interested in being interviewed

**Please tell us your name**

________________________________________________________________

Display this question:

If Would you be interested in taking part in an interview with a researcher from Coventry University? = Yes, I am interested in being interviewed

**Phone number**

________________________________________________________________

Display this question:

If Would you be interested in taking part in an interview with a researcher from Coventry University? = Yes, I am interested in being interviewed

**Email address**

________________________________________________________________
