# Supplementary material for: A mixed methods evaluation of a facilitated research career pathway for nurses, midwives, allied health professionals and healthcare scientists working in the NHS
Source: BMC Med Educ. 2025 Oct 17;25:1438. doi: 10.1186/s12909-025-07982-2 (PMC12532386; doi:10.1186/s12909-025-07982-2)
Supplement: Supplementary file 2 — Supplementary Material 2 [file 12909_2025_7982_MOESM2_ESM.docx]

**Interview schedule – iCAhRE programme participants**

**Current role**

What is your current role?

How long have you been in your current role?

In your current role, do you spend any time on research activity? If yes, how much roughly (percentage)?

**Participation in the iCAhRE programme**

When did you take part in iCAhRE and which levels of the programme did you undertake?

What support has the iCAhRE team/R&D team/Centre for Care Excellence given you before or after starting the programme?

What was your clinical role when you began iCAhRE?

How did you hear about iCAhRE?

What were your reasons for applying for iCAhRE? What did you hope to get out of it?

What were your reasons for participating in (or not participating in) further levels of iCAhRE?

What were your experiences of applying for iCAhRE? (How easy was it? Application process? Support to apply?)

**Experiences of iCAhRE**

What have been your experiences of participating in iCAhRE?

What aspects of iCAhRE were most beneficial to you? What was least beneficial?

Did the iCAhRE programme cover everything you needed it to? Were there things missing?

How relevant was iCAhRE to your own career aspirations?

What have been your experiences of any support provided to you by the iCAhRE team/R&D team/Centre for Care Excellence in relation to your participation in the programme?

Overall, how satisfied have you been with the support and opportunities you have been offered through the iCAhRE programme?

**Learning from iCAhRE**

In what ways has iCAhRE developed your research skills and understanding of research?

As a result of iCAhRE, how confident do you feel to start or continue undertaking clinical research?

Do you think you have gained new skills from participating in iCAhRE that will benefit your current role/your future career? What are these skills?

To what extent has iCAhRE impacted your motivation to start or continue a clinical academic career?

**Impact of iCAhRE**

What have you done as a consequence of participating in iCAhRE?

Prompt (if needed):

Discussed evidence base with patients/colleagues

Questioned own practice more

Searched/critically appraised literature

Used the evidence based to inform my clinical practice

Undertaken audit to improve practice

Undertaken service improvement/quality improvement work

How has iCAhRE had an impact or changed your clinical practice?

What are you doing differently?

Can you give me examples?

How have these changes been beneficial?

Do you have any evidence of benefits to practice?

Have you had any influence on the practice of others? If yes, what impact?

Have you supported others to develop their clinical academic careers? If so, how?

Please tell me about any research activity you have done during/after participating in iCAhRE.

Prompt:

Research projects

Research funding/grants

Presentations and posters

Written for professional magazine

Publications

Awards

(Ask for more details of any of these are mentioned. Tell me more about that?)

What impact has your research activity had? (On patients? On clinical practice? Wider impact?)

What impact has iCAhRE had on your clinical academic career?

Have you changed your role/employment in any way?

**Career trajectory and aspirations**

What are your aspirations for your career? Where do you see yourself in 5-10 years time?

What factors impacted upon your decision to pursue (or not pursue) a clinical academic career?

What helps clinicians pursue a clinical academic career?

What are the barriers?

(If currently pursuing a clinical academic career) What has helped you?

What was a barrier?

What facilitators and barriers do you anticipate in the future?

How do you negotiate the 2 career paths?

What could be done to improve clinical academic career pathways?

**Future recommendations**

What should iCAhRE look like in the future?

Are there any changes that you think should be made?

(If more than one change mentioned) What is the most important change that should be made?

Is there anything else you would like to add?
